# Thirteen is enough: the myosins of *Dictyostelium discoideum *and their light chains

**DOI:** 10.1186/1471-2164-7-183

**Published:** 2006-07-20

**Authors:** Martin Kollmar

**Affiliations:** 1 Abteilung NMR basierte Strukturbiologie, Max-Planck-Institut für Biophysikalische Chemie, Am Fassberg 11, D-37077 Goettingen, Germany

## Abstract

**Background:**

*Dictyostelium discoideum *is one of the most famous model organisms for studying motile processes like cell movement, organelle transport, cytokinesis, and endocytosis. Members of the myosin superfamily, that move on actin filaments and power many of these tasks, are tripartite proteins consisting of a conserved catalytic domain followed by the neck region consisting of a different number of so-called IQ motifs for binding of light chains. The tails contain functional motifs that are responsible for the accomplishment of the different tasks in the cell. Unicellular organisms like yeasts contain three to five myosins while vertebrates express over 40 different myosin genes. Recently, the question has been raised how many myosins a simple multicellular organism like *Dictyostelium *would need to accomplish all the different motility-related tasks.

**Results:**

The analysis of the *Dictyostelium *genome revealed thirteen myosins of which three have not been described before. The phylogenetic analysis of the motor domains of the new myosins placed Myo1F to the class-I myosins and Myo5A to the class-V myosins. The third new myosin, an orphan myosin, has been named MyoG. It contains an N-terminal extension of over 400 residues, and a tail consisting of four IQ motifs and two MyTH4/FERM (myosin tail homology 4/band 4.1, ezrin, radixin, and moesin) tandem domains that are separated by a long region containing an SH3 (*src *homology 3) domain. In contrast to previous analyses, an extensive comparison with 126 class-VII, class-X, class-XV, and class-XXII myosins now showed that MyoI does not group into any of these classes and should not be used as a model for class-VII myosins.

The search for calmodulin related proteins revealed two further potential myosin light chains. One is a close homolog of the two EF-hand motifs containing MlcB, and the other, CBP14, phylogenetically groups to the ELC/RLC/calmodulin (essential light chain/regulatory light chain) branch of the tree.

**Conclusion:**

*Dictyostelium *contains thirteen myosins together with 6–8 MLCs (myosin light chain) to assist in a variety of actin-based processes in the cell. Although they are homologous to myosins of higher eukaryotes, the myosins of *Dictyostelium *should be considered with care as models for specific functions of vertebrate myosins.

## Background

Three kinds of molecular motor proteins have been identified so far: myosins, kinesins, and dyneins [1,2]. While kinesins and dyneins move on microtubule tracks, myosins are the only motors that use the energy of ATP hydrolysis to power movement along actin filaments. The first myosin was identified in skeletal muscle tissue, and subsequently a large number of proteins containing the myosin motor domain have been discovered in eukaryotic cells where they fulfill a variety of cellular functions from cell division, cellular locomotion, vesicle transport to muscle contraction [1].

Myosin proteins are typically divided into three major domains. The motor domain that is usually found at the N-terminus contains the nucleotide and actin binding sites. The neck domain, following the motor domain, consists of a helical segment that binds specific myosin light chains or calmodulin. The target sequence for light chain binding is based on the consensus sequence IQxxxRGxxxR [3] and therefore termed the IQ motif. Myosins may have zero to over 15 IQ motifs in the neck region. The third domain is called the tail domain and contains class specific functional motifs that are responsible for the accomplishment of the different tasks of the myosins in the cell [1]. The classification of the myosins is based on the phylogenetic relation of the myosin motor domains. Altogether, up to 20 myosin classes have been assigned in recent reviews [4,5]. In addition, there are many myosins for which close homologs have not been found and which are therefore termed orphan myosins. Recently, two analyses of myosin proteins describing conflicting findings have been published [6,7]. Both disagree with previously established models of myosin evolution [reviewed in [5]], because of the erroneous data sets and analysis methods used. However, we have performed an exhaustive analysis of 1910 manually annotated myosins from 303 species that will be referred to in the analysis of the *Dictyostelium discoideum *myosins (F. Odronitz and M. Kollmar, submitted).

Different organisms contain only a subset of the classes but in many cases several homologs of the same class. For example, the *Entamoeba histolytica *genome reveals one class-I and one class-II myosin [8], while the *Saccharomyces cerevisiae *genome contains two class-I, one class-II, and two class-V myosins [9]. In contrast, more complex genomes show more diversity. The *Caenorhabditis elegans *genome contains 17 myosins belonging to seven classes, the *Drosophila melanogaster *genome contains 13 myosins belonging to ten classes, and the *Homo sapiens *genome reveals 40 myosins belonging to twelve classes. Plants do not have a large repertoire of classes but many homologs of the class-VIII and class-XI myosins (e.g. *Arabidopsis thaliana *contains 4 class-VIII and 13 class-XI myosin genes).

For the lower eukaryot *Dictyostelium discoideum*, 14 myosins or potential myosins have been reported so far. The first myosin identified, a class-II myosin, has been named mhcA (myosin heavy chain A) and all subsequently discovered myosins where named alphabetical (MyoA-M). For MyoF and MyoH only small fragments of the motor domain have been obtained while for MyoG and MyoL only potential loci have been identified. The class-I myosins (MyoA-E, MyoK) and the class-II myosin have unambiguously been assigned in the past [e.g. [5,10]]. MyoI has been classified as a class-VII myosin [5,11], although the phylogenetic grouping has been very weak, while no similar myosin has been found to MyoM that has therefore been added to the orphan class. The closest homologues found for MyoJ came from the class-XI myosins, a class only containing plant myosins, and it was therefore grouped to them [12,13].

Almost all myosins exist at least as heterodimers by binding of light chains to the neck region. The light chains bind to the IQ motif and have an essential role in stabilizing the neck, so it can function as a rigid lever arm that swings relative to the motor domain to generate movement [14]. In addition, the light chains can be important regulatory sites, either through phosphorylation or by binding of Ca^2+ ^[15]. The light chains belong to the family of calmodulin-related proteins. Most myosins are expected to bind calmodulins, while the class-II myosins always bind two specific calmodulin-related proteins, the essential and the regulatory myosin light chains. Recently, calmodulin-related proteins have been identified that specifically bind to a certain myosin, e.g. a light chain binding to *Toxoplasma gondii *Myo14A [TgMLC-1, [16]], or the light chain binding to *Accanthamoeba castellanii *Myo1C [AcMICLC, [17]]. For *Dictyostelium discoideum*, in addition to the essential and the regulatory myosin light chains two further specific light chains have been discovered. The class-I myosin MyoD binds a light chains that is phylogenetically related the *Ac*Myo1C light chain [MlcD, [18]], and the class-I myosin MyoB forms a heterodimer with a light chain that is unique as it consists of only the half of a calmodulin-related protein [MlcB, [19]].

*Dictyostelium *is one of the most famous model organisms for studying motile processes in cells, especially those related to the actin cytoskeleton. Recently, the question has been raised how many myosins a simple multicellular organism like *Dictyostelium *would need to accomplish all the diverse motility-related tasks [10]. Here, the complete repertoire of myosin family proteins in the slime mold *Dictyostelium discoideum *is presented. The analysis revealed thirteen myosin proteins of which three have not been described so far. The new myosin family members are described and the already published myosins revised and partially reclassified. In addition, all members of the calmodulin-related protein family in *Dictyostelium *have been identified and analyzed to reveal the complete repertoire of myosin light chains.

## Results and discussion

### Identification of *Dictyostelium discoideum *myosins

The TBLASTN search with the motor domain of the *Dictyostelium discoideum *class-II myosin against the *Dictyostelium *genome sequence retrieved the previously identified and described genes and three new myosin genes (Table 1). Small fragments of two of these myosins (Myo1F and Myo5A, former MyoH) have already been obtained in an investigation that combined low-stringency hybridization, physical mapping techniques, and PCR [20], but have not been verified in later studies. The study also revealed two additional loci that were referred to as *myoG *and *myoL*. The analysis of the *Dictyostelium *genome now showed that the *myoG *locus is a real locus, and the corresponding new myosin protein has been named MyoG. However, there is no evidence for further myosin genes and the *myoL *locus has most probably been assigned based on experimental artefacts.

The *Dictyostelium *cDNA database in Japan [21] was searched for the new myosin genes to confirm their expression (exclusion of pseudogenes) and gene structure, as the newly identified *myoF*, *myoH*, and *myoG *genes contain several introns (Table 1). However, the cDNA clones only cover the region around the last of the introns of Myo5A (MyoH). The extremely high AT content of *Dictyostelium *introns and the help of a multiple sequence alignment of over 1700 myosin motor domains (M. Kollmar, unpublished data) nevertheless allowed the unambiguous identification of the introns and, subsequently, the protein coding regions.

The *Dictyostelium *cDNA database also contains at least cDNA fragments for all previously reported myosins. The analysis of the genome sequence and the cDNA data revealed several major discrepancies to the published sequences (Table 2) in addition to many amino acid substitutions. The sequences derived from the genome-sequencing project are without much doubt the correct sequences, because the genome sequence was build on high coverage and is completely in accordance with the cDNA data. Also, the sequences derived from the genome data are in agreement with the multiple sequence alignment while the published old sequences create strange insertions and substitutions. It is very unlikely that the differences are due to strain differences because the AX4 strain, that has been used to create the genome sequence and cDNA libraries, has been derived from the AX2 and AX3 strains used in earlier publications [22].

### Expression pattern of the new myosin genes

The cDNA database is based on libraries of cells obtained from several different stages of the developmental cycle of *Dictyostelium *[23]. One library contains data from clones obtained at the so-called first finger stage (14 h – 16 h) of development. A second library was constructed from vegetatively growing cells. A third library contains clones derived from "sexually competent cells" (cells are cultured in liquid medium in the dark, and are competent for fusion with opposite mating type cells), cells that are roughly equivalent to growth phase cells. The data supposed to contain full-length genes is based on libraries from cells at the following stages of the developmental cycle: axenically growing cells, cells developed on nitrocellulose filter to aggregation stage (8 hours), slug stage (16 hours) and early culmination stage (20 hours). For Myo5A (MyoH) and MyoG cDNA fragments have been obtained in all libraries containing the potentially full-length genes indicating that these two myosins are expressed in all stages of the developmental cycle. However, only one gene fragment has been obtained for Myo1F from the library of full-length genes of axenically growing cells. The cDNA data is not supposed to reveal the complete expression pattern of all proteins at the different developmental stages. But the number of obtained clones and the occurrence in a specific library indicates that Myo1F might not be strongly expressed, and primarily expressed in vegetative cells. These conclusions have of course to be confirmed by further experimental data.

### Classification, nomenclature, and phylogenetic analysis

**Figure 1:**
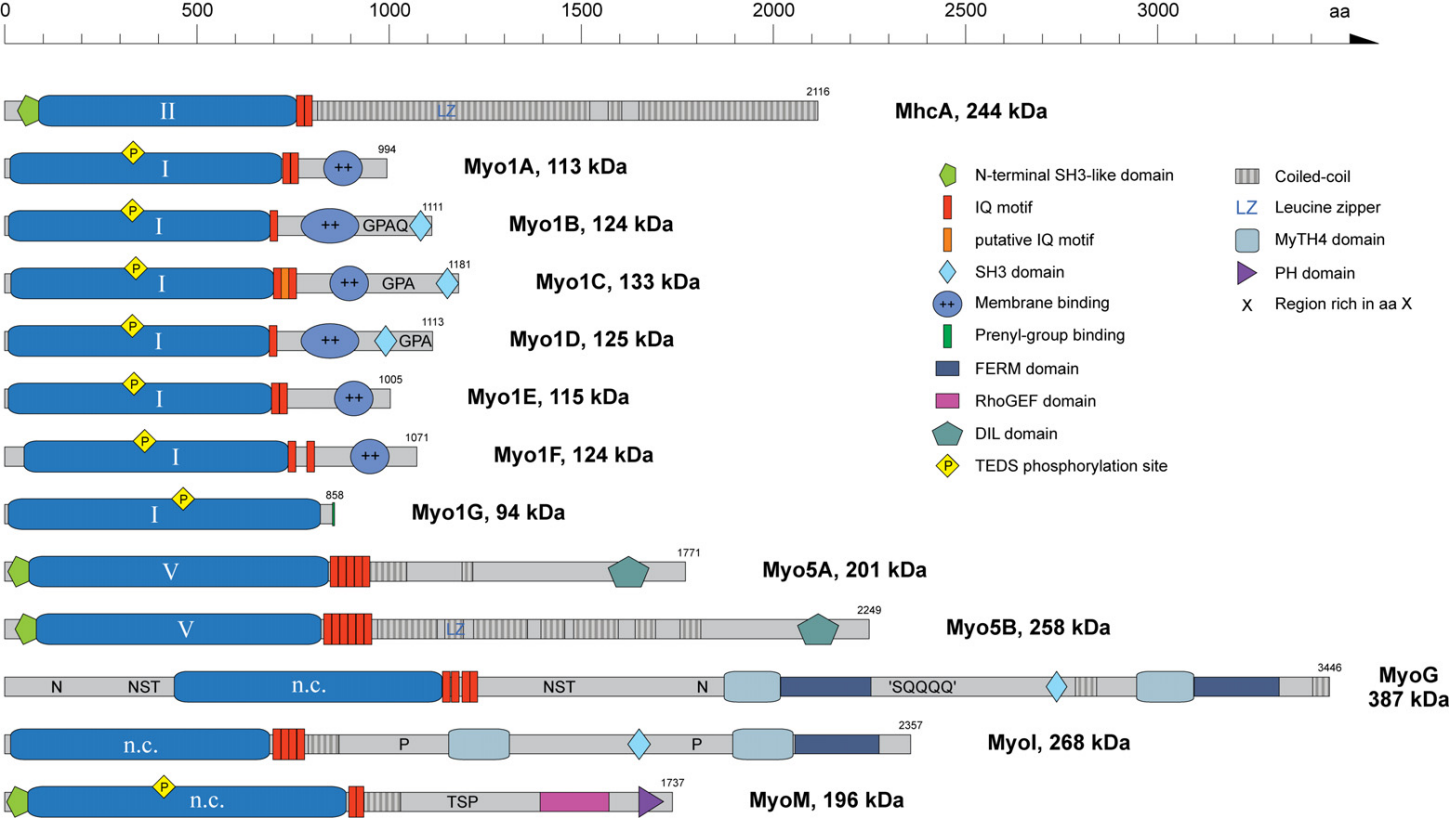
**Schematic diagram of the domain structure of the *Dictyostelium discoideum *myosins**. The class designation is given in the motor domain of the respective myosin in Roman numerals. Orphan myosins have been designated n.c. (not classified). A colour key to the domain names and symbols is given on the right except for the myosin domain that is coloured in blue.

The classification and suggested revised nomenclature of the *Dictyostelium *myosins is summarized in Table 1 and shown in Figure 1. The general nomenclature for myosin proteins uses the term Myo followed by the class number (Arabic numeral) and the variant (Arabic letter). The class-II myosins are exceptions as Mhc or Myh is used as abbreviation (leaving out the class number) followed by the variant designations as either Arabic letters or Arabic numerals. To not severely increase the number of classes, myosins that do not have a homolog in at least one other organism should be referred to as orphans. So far, the *Dictyostelium *myosins have not consistently been named according to that nomenclature. The phylogenetic analysis of over 1700 myosin motor domains (M. Kollmar, unpublished data) together with the completed sequence of the *Dictyostelium *genome now allows a revision. The new nomenclature does not severely change the old names as the class-II myosin stays untouched, the class-I myosins only get the class designation added with the exception of MyoK that will now be referred to as Myo1G, and MyoM is still an orphan myosin and will not be renamed as long as it cannot be grouped to a certain class. The newly identified MyoG (preliminarily named according to its locus) is also an orphan myosin and might be renamed as soon as further homologous myosins are derived. However, the classification of the other myosins, MyoH, MyoJ, and MyoI, is not that unambiguous.

**Figure 2:**
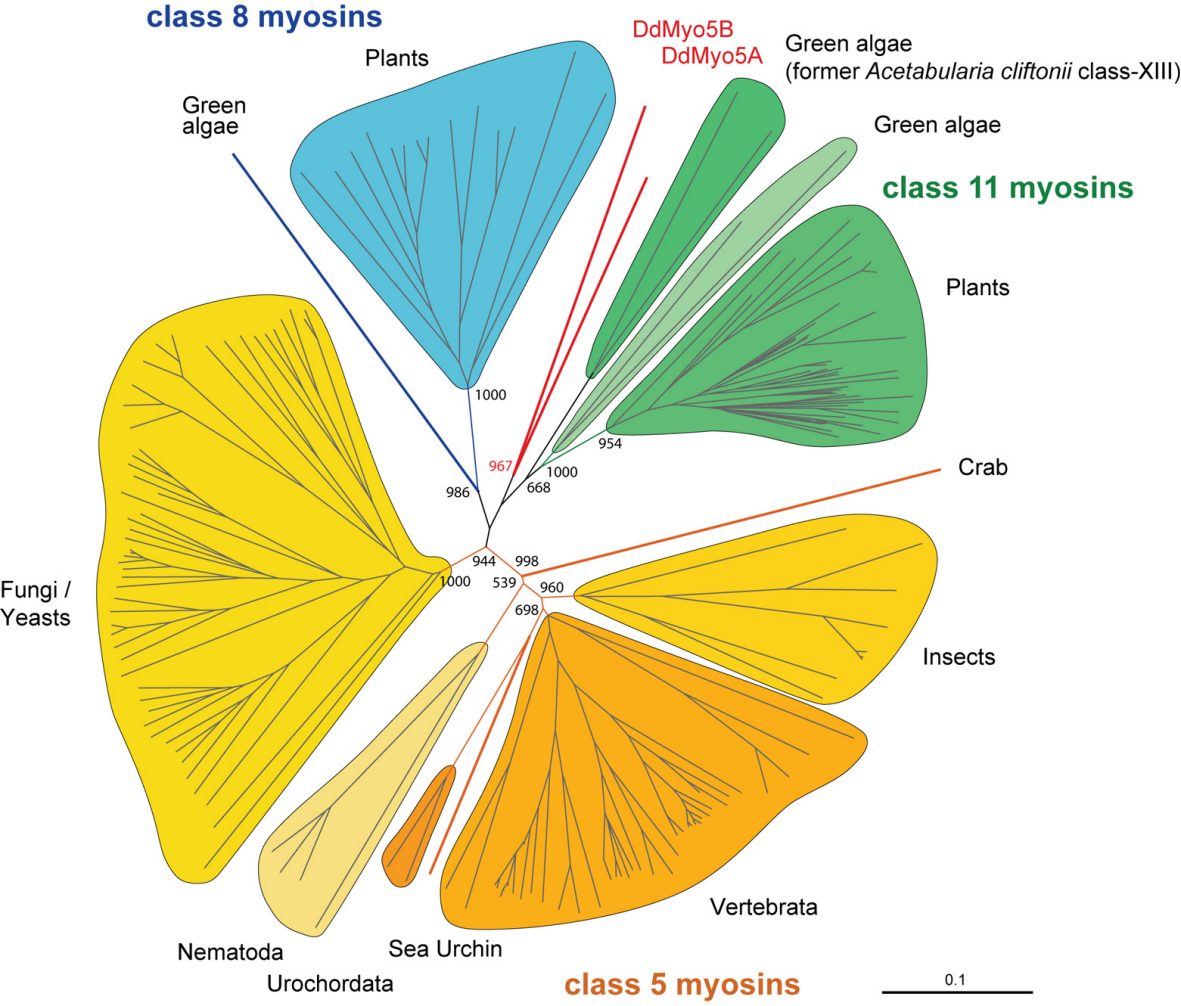
**Phylogenetic tree of 180 motor domains of class-V, -VIII, and -XI myosins (including the former class-XIII myosins)**. Amino acid sequences of the motor domains were aligned in a structure-guided manual alignment process. Support values for each internal branch were obtained by 1,000 bootstrap steps. The values for the innermost branches are given. The scale bar corresponds to 0.1 estimated amino acid substitutions per site. See additional file 1: SuppMat1 for the complete tree containing all internal labels.

A phylogenetic tree of 180 myosins of the classes V, VIII, and XI (including the former class-XIII myosins) does not group MyoH and MyoJ to any of the already assigned classes (Figure 2, additional file 1). Instead of assigning these myosins a new class, as it happened in the past e.g. for the classification of the *Acetabularia cliftonii *class-XIII myosins, I suggest to name MyoH and MyoJ Myo5A and Myo5B, respectively. Both myosins have a similar domain organisation as the class-V and the class-XI myosins. But because *Dictyostelium *separated from the Fungi/Metazoa lineage after the separation of the plants, MyoH and MyoJ should rather be referred to as class-V than class-XI myosins. This classification is supported by the analysis of the over 1700 myosins that revealed MyoH and MyoJ to be closer related to the class-V myosins than to the plant myosins (M. Kollmar, unpublished data).

**Figure 3:**
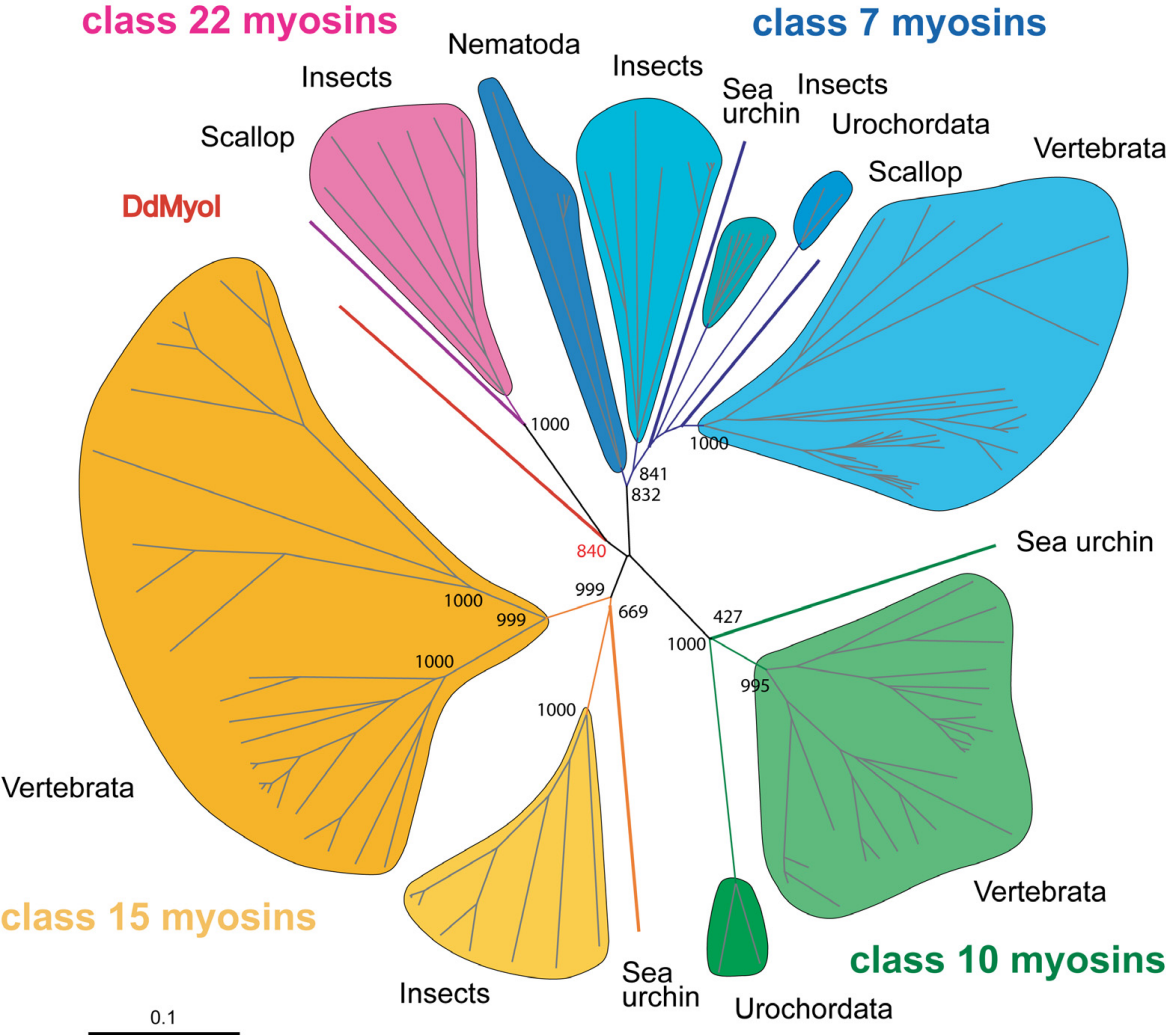
**Phylogenetic tree of 126 motor domains of class-VII, -X, -XV, and -XXII myosins**. Amino acid sequences of the motor domains were aligned in a structure-guided manual alignment process. Support values for each internal branch were obtained by 1,000 bootstrap steps. The values for the innermost branches are given. The scale bar corresponds to 0.1 estimated amino acid substitutions per site. See additional file 2: SuppMat2 for the complete tree containing all internal labels.

**Figure 4:**
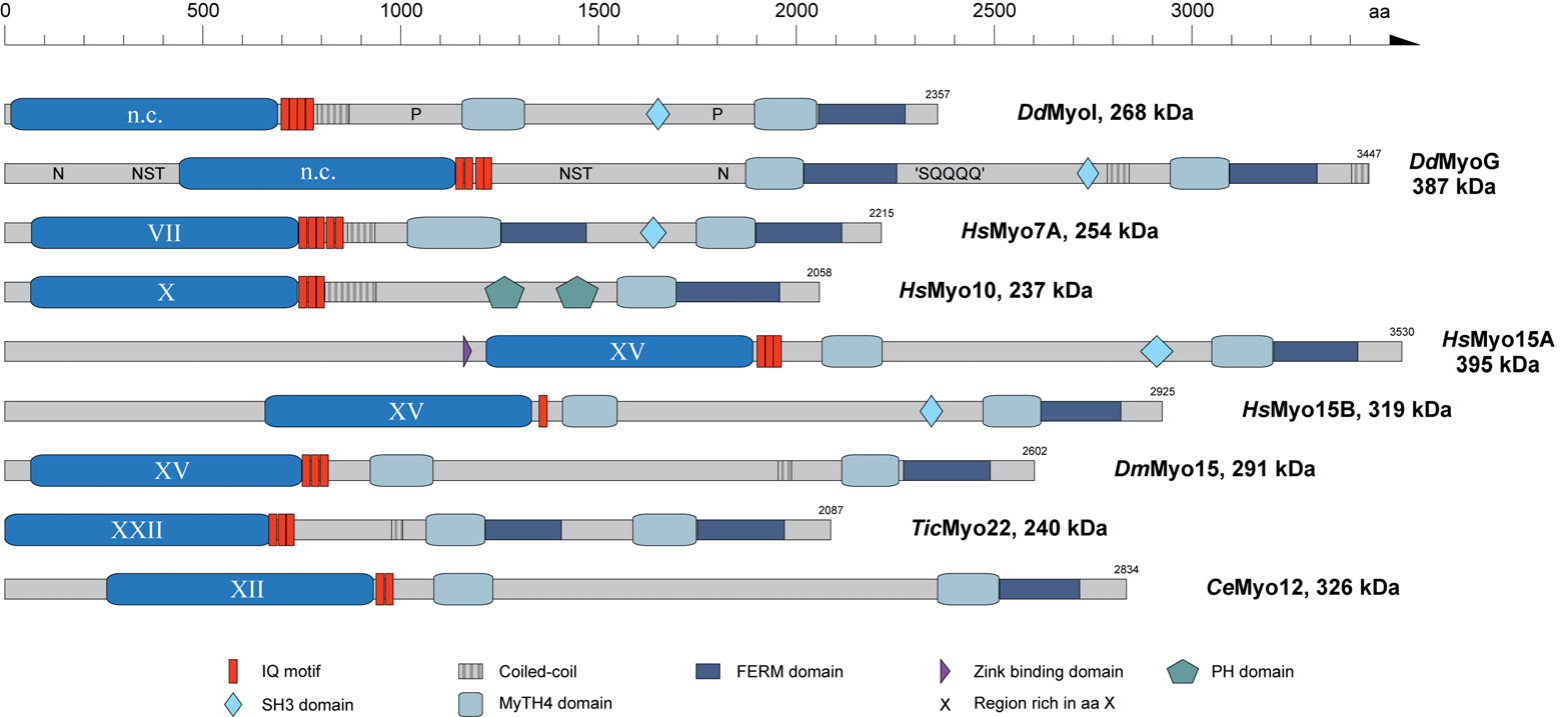
**Domain organisation of examples of class-VII, -X,-XV, and -XXII myosins**. The class designation is given in the motor domain of the respective myosin in Roman numerals. A colour key to the domain names and symbols is given on the bottom except for the myosin domain that is coloured in blue. For comparison, a myosin of class-XII is shown because its tail also contains MyTH4 and FERM domains, although class-XII myosins phylogenetically do not group at all to the other classes shown.

MyoI does not group to any of the designated classes containing myosins with MyTH4/FERM domains as is shown by the phylogenetic analysis of 126 myosin motor domains of classes VII, X, XV, and XXII (Figure 3, additional file 2). It does not group to the class-XXII myosins although it branches from the class-XXII myosins in the tree shown. The reason is that the branching occurs very close to the separation point of the other classes, and in phylogenetic trees including all classes MyoI also often branches very early from the class-X myosins. A close view at the protein sequence in the multiple sequence alignment shows that MyoI shares several class specific features off all four classes that prevent a better classification. In addition to the motor domain sequence, the domain organisation of MyoI is unique compared to members of the other classes (Figure 4). In contrast to class-VII myosins, MyoI does not have an N-terminal SH3-like domain, it has four instead of five IQ-motifs and it misses the first FERM domain. The tails of class-X myosins are different to that of MyoI as they are characterised by two consecutive PH (pleckstrin homology) domains followed by the MyTH/FERM tandem domain. The domain organisation of the class-XV myosin tails is similar to that of MyoI except that the mammalian myosins have a very long N-terminal domain, while the insect myosins miss the SH3 domain. Like MyoI, the class-XXII myosins do not have an N-terminal domain, but their tail domain is different containing two complete MyTH/FERM tandem domains but no SH3 domain. Thus, MyoI cannot be grouped to any of the already designated classes and should be considered as an orphan myosin. This implicates that MyoI cannot be considered as a specific model for class-VII myosins as it has been suggested earlier [11,24]. The *Dictyosteliida *diverged before the evolution of the Metazoa. MyoI therefore rather resembles a common ancestor of the four classes instead of grouping to one of them.

### Domain structure of the known myosins

**Figure 5:**
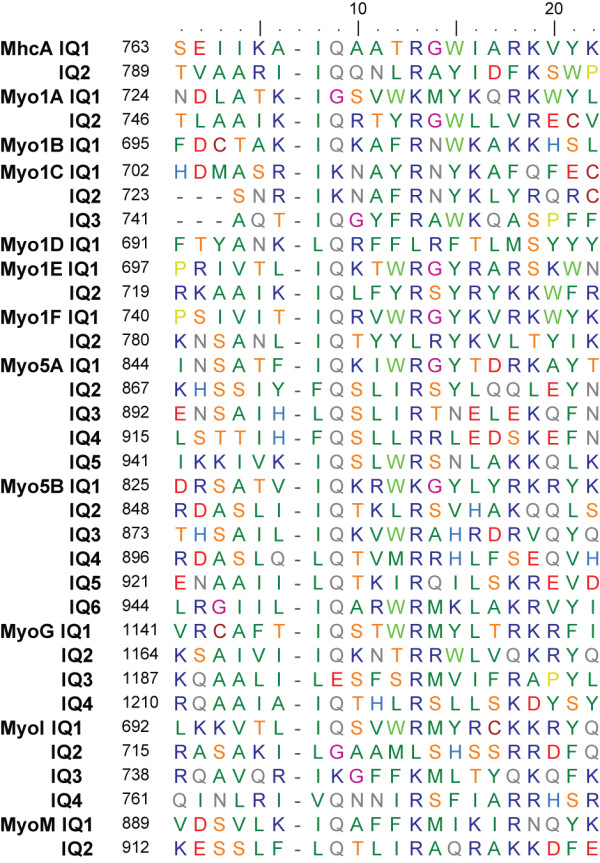
**Alignment of the putative IQ motifs of the *Dictyostelium *myosins**. The alignment shows the IQ motifs of the myosins. The numbers at the beginning indicate the position in the full-length sequence. The gap has only been introduced to facilitate the identification of the IQ residues.

Most of the tail domains of the known myosins have already been described and functionally analysed in some detail [25,26]. While there is agreement on the determination of the larger tail domains like the class-I myosin membrane-binding and SH3 domains, or the Myo5B (MyoJ) DIL (*dilute*) domain, there are contradicting predictions of the IQ motifs. The IQ motif is a short sequence motif of alpha-helical structure that is able to bind calmodulin or calmodulin-like proteins. IQ motifs have been predicted in the past by using the general pattern IQxxxRGxxxR [3]. The multiple sequence alignment of the whole myosin family now allows a revision of the motif (M. Kollmar, unpublished data). According to the revised motif several IQ motifs are found in the *Dictyostelium *myosins that have not been recognised before (Figure 1 and Figure 5). The starting isoleucine in the motif is often substituted by other large hydrophobic amino acids. The glutamine at the second position is mainly conserved, except for some cases where it can be substituted by lysine, glycine, or glutamate. A very important position is the residue before the first arginine, that is almost always a large hydrophobic amino acid, in most cases an aromatic one. The first arginine is also highly conserved, except for a few cases where it is substituted by lysine, leucine, or isoleucine. The following glycine is not very conserved in IQ motifs of myosin tails. The position after that glycine is mainly occupied by large hydrophobic amino acids, in most cases aromatic residues, but histidines, asparagines, and glutamines are also found. The second arginine of the initial motif is also not very conserved. Using the revised motif, no IQ motif is predicted for Myo1G, one is predicted for Myo1B and Myo1D, and two are predicted for the other class-I myosins. Myo1C might contain a third IQ motif but then the packing of the light chains would be relatively dense. However, Myo1B binds a light chain that consists of only two EF-hand motifs [half the size of a normal calmodulin-related protein, [19]]. Two similarly small myosin light chains could easily bind to the two closely located IQ motifs of Myo1C. According to the new motif description MyoI is now predicted to contain four IQ motifs in contrast to earlier predictions of three IQ motifs [11], and Myo5B (MyoJ) contains six. The domain compositions of the newly identified myosins are described in more detail below.

### Domain structure of the newly identified and analysed myosins

#### Myo1F

This myosin is the seventh class-I myosin found in the *Dictyostelium *genome. Based on the short fragment of the motor domain which has been obtained in an earlier PCR-based screen of the genome [27] it was already supposed to group to this subfamily. Its motor domain sequence is most similar to that of Myo1E. Unlike the other class-I myosins that contain only small N-terminal extensions to the motor domain of 8 to 15 residues it has an N-terminal domain of 50 amino acids. However, based on its sequence this small domain is unlikely to fold into a similar structure like the N-terminal domain of class-II myosins. Directly following the motor domain, Myo1F has two consecutive IQ motifs that are strong indicators for binding of calmodulin or a calmodulin-like myosin light chain. A short coiled-coil region has been predicted for the small region between the two IQ motifs. The short distance of only about 20 residues, however, makes it unlikely that Myo1F would be able to dimerize in that area. The remainder of the tail is similar to those of Myo1A or Myo1E predicted to be all α-helical. In the centre of the tail there is a domain rich in basic residues and it is therefore outlined as membrane binding domain in analogy to those of the other class-I myosins. Whether this region really binds to membranes has to be shown.

#### Myo5A (former MyoH)

Myo5A seems to be the smaller brother of Myo5B (MyoJ). Both are phylogenetically closely related and show similar domain organisations. Altogether, Myo5A (MyoH) is 480 aa shorter in length. It also has an N-terminal SH3-like domain, but the N-terminal extension is not as long as for Myo5B (MyoJ). In contrast to Myo5B (MyoJ), it has three additional long insertions into surface loops of the motor domain that are not observed in any other *Dictyostelium *myosin sequence. It has five instead of six IQ motives for binding of light chains, and the coiled-coil region is predicted to be considerably shorter than that of Myo5B (MyoJ). Except for the DIL domain at the C-terminus of the tail there is no further sequence similarity between the two Myo5 tail sequences.

#### MyoG

MyoG does not group to any of the class-VII, class-X, class-XV, or class-XXII myosins, or any other myosin, and is therefore designated an orphan myosin. MyoG is one of the longest myosins of the whole family. It has an N-terminal domain of 440 residues that does not show any homology do other proteins. The sequence in the N-terminal domain contains long stretches of consecutive asparagines and serines that are typical for many *Dictyostelium *proteins [28]. The head domain is followed by four IQ motifs. The C-terminal tail is characterised by two MyTH4/FERM tandem domains that are separated by a long region containing an SH3 domain and a short predicted coiled-coil region. The regions between these recognised domains also contain many stretches of consecutive polar residues. An outstanding case is the nine-fold consecutive repeat of the motif 'SQQQQ'. The C-terminal end of the tail contains a second predicted coiled-coil region. The coiled-coil domains in myosins are normally located directly behind the IQ motifs and are responsible for dimerisation. MyoG might also exist as a dimer *in vivo*, but the heads are not expected to move on actin filaments in a similar hand-over-hand mechanism as has been found for other myosin dimers like class-V myosins [29].

### Structural features of the myosin motor domains

**Figure 6:**
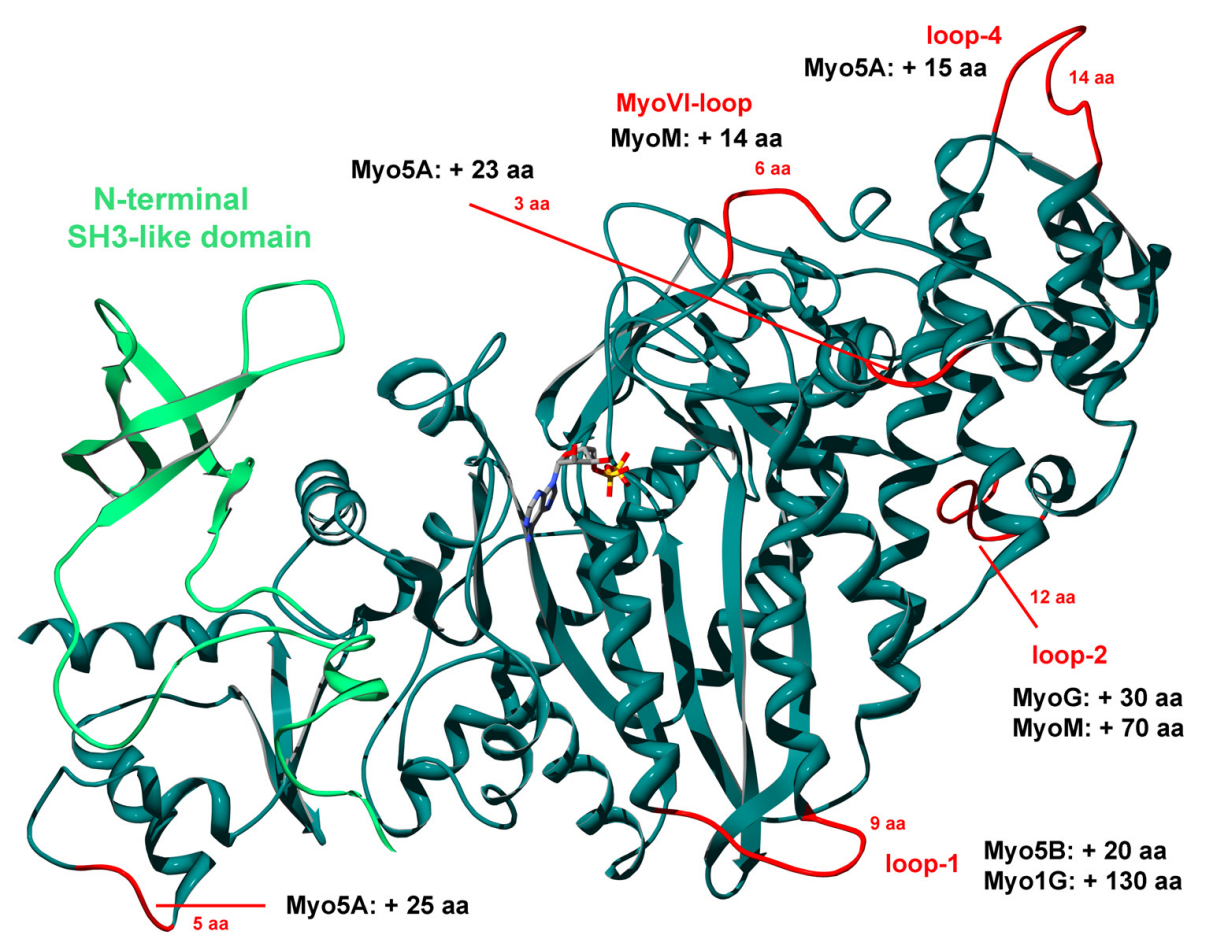
**Structure of the motor domain of *Dictyostelium *MhcA**. Structure of the motor domain of *Dictyostelium *MhcA (PDB: 1g8x) highlighting loops for which some of the *Dictyostelium *myosins have long insertions. The red numbers indicate the length of the loops for MhcA. The approximate lengths of the insertions are given for the respective myosins.

The myosins of *Dictyostelium *contain several protein specific extensions to surface loops of the motor domain of class-II myosin (Fig. 6). The most prominent loop-extension is the insertion of ~130 amino acids into loop-1 of Myo1G. Except for members of an arthropoda specific myosin class, that contain loop-1 extensions of up to 300 amino acids [30], this is by far the longest loop-1 of all myosins. Myo5B (MyoJ) also contains a considerably longer loop-1 (20 residues in addition to loop-1 of MhcA). Loop-1 has been implicated in influencing access to the nucleotide-binding site. It has been shown in an analysis of chimeric loop-1 mutants of smooth muscle myosin that the mobility of this loop correlates to the rate of ADP release [31]. Larger and more flexible loops resulted in faster rates of ADP release. For class-V myosins, the rate-limiting step in the catalytic cycle is ADP release [32], a necessity for these myosins to walk over long distances along the actin filaments without detaching. Based on these results, Myo5B (MyoJ) might be a very unconventional class-V myosin with a fast rate of ADP release. It will be very interesting to see whether Myo5B (MyoJ) is still a long-distance cargo transporting myosin as the other homologs of the class.

Loop-2 has been shown to be involved in both weak and strong binding interactions with actin [33]. According to this study, especially positively charged residues strengthen the binding to actin. MyoG and MyoM contain long extensions of loop-2 compared to MhcA, but the sequences contain mainly glycines, prolines, and polar amino acids. Thus, both myosins are not expected to have considerably different actin-binding properties. Loop-4 is the loop that is furthest removed from the actin surface as has been suggested from actomyosin models derived from electron microscopy. Except for some class-I, the insect class-V, some class-XVII, and some apicomplexa myosins, almost all myosins have a loop-4 of similar length. The loop has been suggested to be involved in either interactions with actin or regulatory proteins that are bound to actin [34]. For the class-I myosin myr1 from rat, it has been shown that a head fragment localizes to the same highly dynamic actin structures at the cell cortex as the full-length construct and not to the actin filaments that are regulated by tropomyosin [35]. An extended loop-4 might be responsible for this localisation as it might hinder binding to the tropomyosin stabilized less dynamic actin filaments as they occur in stress fibers. Myo5A (MyoH) contains one of the longest loop-4 of all myosins and might therefore only bind to the dynamic actin structures at the cell cortex and not to actin structures that are stabilized by other proteins.

MyoM has an extended loop at the same position where the class-VI myosins have one of their prominent insertions. This loop has been suggested to affect nucleotide binding by changing the conformation of a following loop [36]. This loop therefore protrudes within the nucleotide-binding pocket, resulting in a decrease in nucleotide accessibility. The functions of the other two surface loops, for which Myo5A (MyoH) has long extensions, have not been analysed so far.

### Phylogenetic analysis of calmodulin-like proteins in *Dictyostelium*

**Figure 7:**
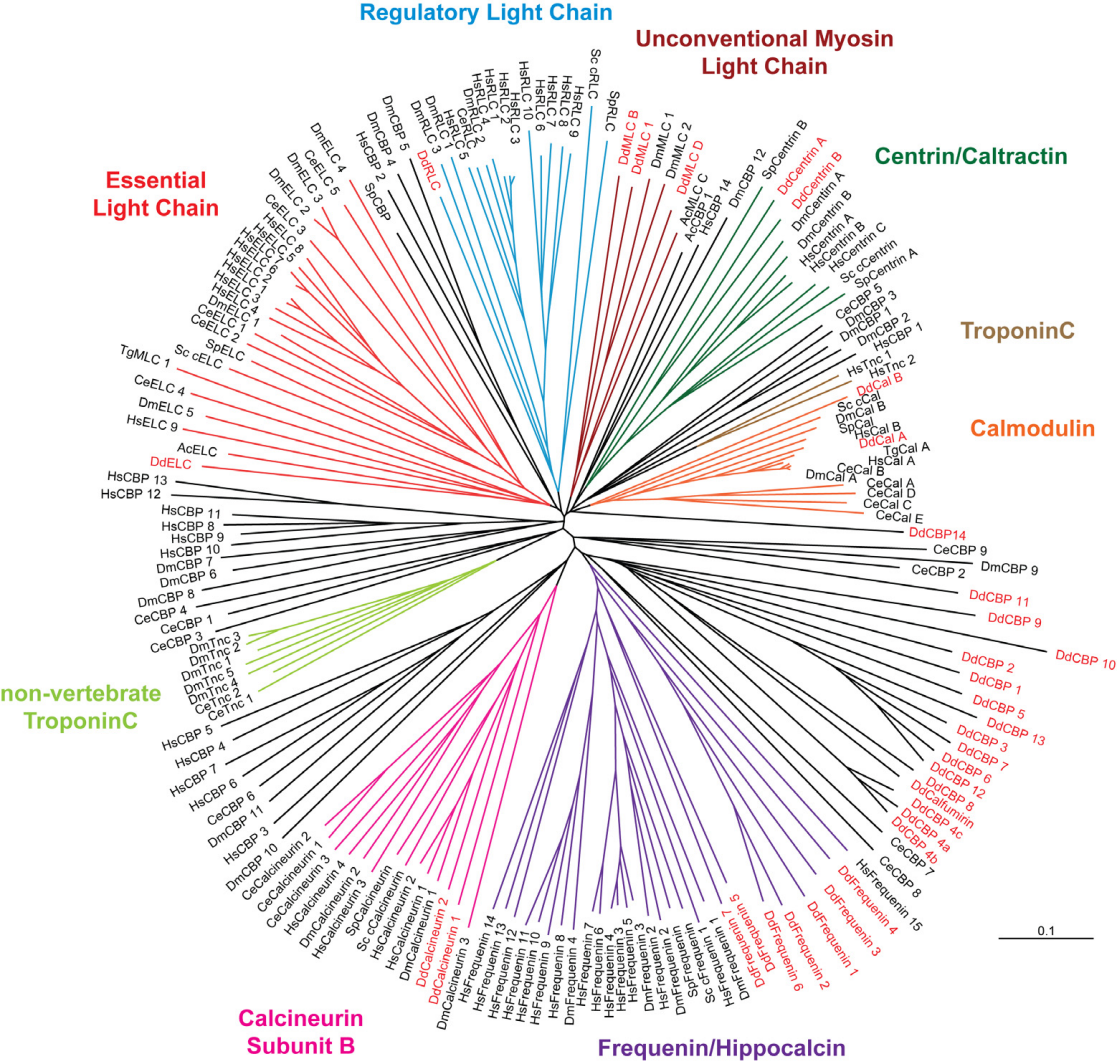
**Phylogenetic tree of calmodulin-related proteins**. Amino acid sequences of the calmodulin-related proteins were aligned manually. Support values for each internal branch were obtained by 1,000 bootstrap steps. The scale bar corresponds to 0.1 estimated amino acid substitutions per site. See additional file 3: SuppMat3 for the complete tree containing all internal labels.

An iterative TBLASTN search of the *Dictyostelium *genome data, starting with the sequence of *Dictyostelium *calmodulin A (*Dd*CalA), revealed 35 CBPs (calcium-binding protein) that exclusively contain EF-hands motifs (Table 3). While 32 CBPs contain four EF-hands, three contain only two EF-hand motifs (MlcB, MLC-1, and CBP10). 22 CBPs have already been described in the literature. To classify the remaining 13 CBPs and to identify those that could potentially function as myosin light chains, the *Dictyostelium *EF-hand proteins were compared with EF-hand containing proteins from *Saccharomyces cerevisiae, Schizosaccharomyces pombe, Caenorhabditis elegans, Drosophila melanogaster, Homo sapiens*, and some specific myosin light chains from other organisms. The obtained 176 calmodulin-related proteins were manually aligned and a phylogenetic tree created (Figure 7, additional file 3).

According to the phylogenetic tree, 16 of the *Dictyostelium *CBPs (CBP1-13, calfumirin) do not belong to an already named class but are closely related to the frequenins. CBP4c is very similar to CBP4a and CBP4b, but the N-terminus could not be identified. CBP13 is a protein fragment, missing the N-terminus as well as the C-terminus, and is very similar to CBP12. CBP4c and CBP13 are therefore most probably pseudogenes. Those members of this group, that have already been identified, belong to developmentally regulated genes. Their distinct spatial expression patterns suggested that they might be involved in morphogenesis [37]. Seven of the *Dictyostelium *CBPs belong to the frequenin class. The members of the frequenin class are also highly developmentally regulated. An interaction with a myosin family protein has not been described in the literature for any member of this group of any organism. *Dictyostelium *also contains two centrins and two proteins grouping to the calcineurin family.

So far, only the ELC and RLC myosin light chains as well as calmodulin subfamily proteins have been shown to bind to the IQ motifs of myosins. The exceptions are the myosin light chain of *Toxoplasma gondii *Myo14A [TgMLC-1, [16]], the light chain of *Accanthamoeba castellanii *Myo1C [AcMIMLC, [17]], and, identified in *Dictyostelium*, a light chain of Myo1D that is phylogenetically related the *Ac*Myo1C light chain [MlcD, [18]], and a light chain that binds to Myo1B [MlcB, [19]]. Next to MlcD and MlcB, *Dictyostelium *contains one ELC and one RLC myosin light chain that bind to MhcA. For *Saccharomyces cerevisiae *it has been shown that the ELC (also termed Mlc1p) not only binds to the class-II myosin, but also to the class-V myosin Myo2p. Chicken myosin-5A even binds two different ELCs next to calmodulin. Therefore, the *Dictyostelium *ELC is also expected to participate in binding to the class-V myosins. *Dictyostelium *also contains two members of the calmodulin subfamily that have not specifically been shown to bind to myosins, but are highly expected to in accordance with results obtained for other organisms. The analysis also revealed another CBP containing two EF-hand motifs that is most closely related to MlcB. It is therefore also highly expected to bind to a myosin, and termed MLC-1. As Myo1B and Myo1D bind a specific light chain each, and Myo1C is the closest homolog of the remaining class-I myosins, MLC-1 might be the specific light chain for Myo1C, but this has of course to be proven by biochemical experiments.

CBP14 does not group to any specific class but is more similar to the members of the calmodulin/ELC/RLC part of the phylogenetic tree then to the developmentally related CBPs. If it were able to function as a myosin light chain, then it would be the founding member for another specific myosin light chain class.

## Conclusion

The analysis of the *Dictyostelium discoideum *genome revealed thirteen members of the myosin family of which three have not been described before. The phylogenetic analysis of their motor domains placed seven myosins to the class-I myosins (Myo1A to Myo1G, Myo1F is a new member), one to the class-II myosins (MhcA), and two, of which Myo5A (MyoH) has newly been identified, to the class-V myosins. Three myosins (MyoG, MyoI, and MyoM) do not have a close homolog in any other organism and could therefore not be classified. In contrast to previous analyses, an extensive comparison with 126 class-VII, class-X, class-XV, and class-XXII myosins now showed that MyoI does not group into any of these classes and can not be used as a model for class-VII myosins. The third new myosin has been named MyoG. It contains an N-terminal extension of over 400 residues, and a tail consisting of four IQ motifs and two MyTH4/FERM tandem domains that are separated by a long region containing an SH3 domain. Although its tail organisation is similar to that of class-VII myosins, the motor domain of MyoG does not group into any existing class.

Four specific myosin light chains have been identified so far (ELC, RLC, MlcB, MlcD) next to two calmodulins. The analysis of the genome revealed another protein containing two EF-hand motifs that is closely related to MlcB. Based on its phylogenetic relationship it is highly expected to be a myosin light chain. A further calmodulin-related protein, termed CBP14, phylogenetically groups to the ELC/RLC/calmodulin branch of the tree and might therefore also be a myosin light chain, although it does not have a close homolog in other model organisms.

## Methods

### Identification of *Dictyostelium *myosins and calmodulin related proteins

The full-length sequences of ten of the thirteen *Dictyostelium *myosins have been reported in the literature (Table 1). Partial sequences of Myo1F (MyoF) and Myo5A (MyoH) have already been reported [27] and were used as basis for the manual assembly of the genes from clones published by the Dictyostelium Genome Sequencing Project. These genes are in consistence with the assembly of the recently published genome [28]. The two sequences, as well as MyoG that has also been derived from genomic data [38], are therefore predicted sequences that have not been verified by complete cDNAs. The Japanese cDNA project [21,23] includes only small parts of these new myosins, which are, however, consistent with the predicted sequences. The Japanese cDNA project also includes at least fragments of all other myosins. No additional myosin genes have been found in the genome of *Dictyostelium*, and thus the reported "*myoL*" gene locus [27] might have originated from experimental artefacts.

The *Dictyostelium *calmodulin-related genes have been identified in an iterated TBLASTN search of the completed *Dictyostelium *genome starting with the protein sequence of CalA. Thus, all solely EF-hand motif-containing proteins have been collected (Table 3). The predicted sequences have been verified by searches against the Japanese cDNA database [23]. The EF-hand containing proteins from *Saccharomyces cerevisiae, Schizosaccharomyces pombe, Caenorhabditis elegans, Drosophila melanogaster*, and *Homo sapiens *have been obtained in iterative TBLASTN searches of the corresponding genomes. Specific myosin light chains from other organisms have been obtained from the protein database at NCBI.

### Building trees

The complete analysis of the myosin motor domains derived from the NCBI non-redundant database and the EST and genomic sequences of more than 270 eukaryotes will be published elsewhere (Kollmar, unpublished data). All these myosin sequences, including the *Dictyostelium *myosins, together with their accession numbers and additional references will be accessible through the newly designed CyMoBase [[39], F. Odronitz and M. Kollmar, submitted]. The database comprises over 1700 myosin sequences (Feb. 2006) that have been used for the phylogenetic classification of the *Dictyostelium *myosins. The underlying phylogenetic tree has been built of a structure-guided multiple sequence alignment. The phylogenetic trees of the class-V/-VIII/-XI and the class-VII/-X/-XV/-XXII myosins have been constructed from corresponding sequences of this alignment. All phylogenetic trees are unrooted and were generated using neighbour joining and the Bootstrap (1,000 replicates) method as implemented in ClustalW [standard settings, [40]] and drawn by using TreeView [41].

The phylogenetic tree of the calmodulin-related proteins has been calculated based on a manual sequence alignment. The manual sequence alignment has been improved by iteratively creating phylogenetic trees and adjusting the alignment. The resulting phylogenetic tree is unrooted and was generated using neighbour joining and the Bootstrap (1,000 replicates) method as implemented in ClustalW and drawn by using TreeView.

### Domain and motif prediction

Protein domains were predicted using the SMART [42,43] and Pfam [44,45] web server. The prediction of protein motifs (coiled coils, leucine zipper, prenyl-group binding motifs) is mainly based on the results of the predict-protein server [46,47]. The IQ-motifs and N-terminal domains were predicted manually based on the homology to similar domains of other myosins included in the multiple sequence alignment of the myosins. The recognition motifs included in the SMART and Pfam databases are too restrictive, as the motifs have been created based on the small datasets available some years ago.

## Abbreviations

SH3, *src *homology 3; PH, pleckstrin homology; DIL, *dilute*; FERM, band 4.1, ezrin, radixin, and moesin; MyTH4, myosin tail homology 4; RhoGEF, Rho GDP/GTP exchange factor; CBP, calcium-binding protein; MLC, myosin light chain; ELC, essential light chain; RLC, regulatory light chain; Chr, chromosome.

## Authors' contributions

M.K. performed all analyses and wrote the manuscript.

## Supplementary Material

Additional File 1 Complete phylogenetic tree of the class-V, -VIII, and -XI myosins. Click here for file

Additional File 2 Complete phylogenetic tree of the class-VII, -X, -XV and -XXII myosins. Click here for file

Additional File 3 Complete phylogenetic tree of the calmodulin related proteins. Click here for file

## Figures and Tables

**Table 1:** Members of the myosin gene superfamily in *D. discoideum*.

Protein name	Gene name	Size in amino acids	Class	No. of introns	Chr.	Gene accession (GeneBank)^a^	Protein accession (GenBank)^b^	dictyBaseID (DDB...)	References
Myo1A	*myoA*	994	1	2	3	S73909 AAFI01000066	AAB20711 EAL67246	0215392	[27]
Myo1B	*myoB*	1111	1	2	5	M26037 AAFI01000196	AAA33229 EAL62866	0191351	[48]
Myo1C	*myoC*	1181	1	1	2	L35323 AAFI01000039	AAC37427 EAL69121	0215355	[49]
Myo1D	*myoD*	1113	1	2	2	L16509 AAFI01000030	P34109 EAL69474	0191347	[50]
Myo1E	*myoE*	1003	1	3	5	L06805 AAFI01000183	AAA33201 EAL63071	0216200	[51]
Myo1F	*myoF*	1071	1	1	5	AAFI01000198	EAL62822	0220021	
Myo1G	*myoK*	858	1	2	2	AF090534 AAFI01000027	AAD47904 EAL70180	0185086	[52, 53]
MhcA	*mhcA*	2116	2	0	4	M11938 AAFI01000135	AAA33227 EAL64202	0191444	[54]
Myo5A	*myoH*	1771	5	3	5	AAFI01000209	EAL62703	0188441	
Myo5B	*myoJ*	2249	5	2	2	U42409 AAFI01000020	AAA85186 EAL71208	0185050	[12, 13]
MyoG	*myoG*	3446	n.c.	3	2	AAFI01000034	EAL69262	0167014	
MyoI	*myoI*	2357	n.c.	3	2	L35321 AAFI01000027	AAF06035 EAL70120	0185049	[11]
MyoM	*myoM*	1737	n.c.	2	6	AF090533 AAFI01000267	AAD47903 EAL61255	0191100	[53, 55]

a The upper numbers refer to the published cDNA derived sequences, the lower numbers to the genomic sequences.

b The upper numbers refer to the protein sequences translated from cDNA, the lower to the sequences translated from genomic DNA.

**Table 2:** Differences in the sequences of previously published genes and the genes obtained from the genome sequencing project.

Protein name	Sequence differences. The first residue/sequence is from the genome project, the numbers indicate the position in the protein sequence.	Example cDNA sequences covering the revised residues.
Myo1A	^3^T instead of E;^211^SSEEE^215 ^instead of HQG; ^239^CFTA^242 ^instead of WFSLP; ^270^EI instead of KS; ^583^R instead of S; ^638^L instead of S; ^809^ATLKR^813 ^instead of DALPS; ^819^AVLQL^823 ^instead of DCSSKR	AHM490, VFH810, AHH186, VHP545
Myo1B	^207^A instead of R; ^365^R instead of K	AFK241, AHL184, AHE272
Myo1C	^479^GADQKLLQSIAVCKSNPHFDTR^500 ^instead of VPIKSYFNPLPFVNQIHISILV ^813^SSKSQVMVHPI^823 ^instead of LQISSHGTPN	SLH381, SHG368, AHF290
Myo1D	^613 ^no insertion of FGRI; ^694^A instead of R; ^837^T instead of R	AFJ116, VFN526
Myo1E	^26^E instead of D; ^48^T instead of R; ^77^M instead of I; ^137^LD instead of IRFQ; ^215^D instead of N; ^371^IINCTTEKGP instead of LSIVHREGT; ^427^VRE instead of K, ^440^N in addition, ^498^I instead of N; ^604^V instead of D; ^681^N instead of I; ^683^T instead of R; ^735^K instead of N; ^763^H instead of D; ^828^S instead of W; ^889^K instead of N; ^979^NQ instead of KE	VHK623, SHL808, AHG510,
Myo5B (MyoJ)	^191^F instead of L; ^284^T instead of A; ^291^R instead of G; ^550^K instead of N; ^865^QQ instead of HH; ^1041^K instead of N; ^1389^Q instead of P	AHL227

**Table 3:** Members of the myosin light chain, calmodulin and calmodulin-related gene superfamily in *D. discoideum*.

Protein name	Gene name	Size in aa	No. of introns	Chr.	Gene accession (GenBank)	Protein accession (GeneBank)	dictyBaseID (DDB...)	References, comments
ELC	*mlcE*	150	1	3	AAFI01000052	EAL68102	0214813	[56, 57]
RLC	*mlcR*	161	2	2	AAFI01000032	EAL69338	0185146	[58]
MlcB	*BC5V2_0_01417*	73	2	5	AAFI01000224	EAL62399	0188713	[19]
MlcD	*mlcD*	147	2	3	AAFI01000052	EAL68131	0214812	[18]
MLC1	*BC5V2_0_01163*	80	2	5	AAFI01000214	EAL62662	0219456	
CalA	*calA*	152	3	3	AAFI01000060	EAL67642	0214955	[59]
CalB	*calB*	149	2	1	AAFI01000011	EAL71901	0191193	[60]
Calcineurin1	*BC4V2_0_01538*	183	3	4	AAFI01000129	EAL64441	0218775	
Calcineurin2	*cnbA*	180	3	1	AAFI01000009	EAL73175	0191204	
CBP1	*cbpA*	156	1	2	AAFI01000042	EAL68881	0185026	[61]
CBP2	*CBP2*	168	2	1	AAFI01000009	EAL73181	0191196	[62]
CBP3	*cbpC*	166	3	4	AAFI01000093	EAL65573	0191405	[63], cluster with *cbpC*, *cbpG*, *cbpF*, CBP14
CBP4a	*cbpD1*	162	3	4	AAFI01000087	EAL65815	0219925	[64]
CBP4b	*cbpD2*	162	3	4	AAFI01000086	EAL65870	0191416	[64]
CBP4c	*BC4V2_0_01277*	145	2	4	AAFI01000124	EAL64575	0186519	pseudogene?
CBP5	*cbpE*	180	2	2	AAFI01000027	EAL69942	0185183	[37]
CBP6	*cbpF*	174	3	4	AAFI01000093	EAL65572	0191383	[37]
CBP7	*cbpG*	169	3	4	AAFI01000093	EAL65571	0191382	[37]
CBP8	*cbpH*	165	2	5	AAFI01000181	EAL63115	0191153	[37]
CBP9	*cbpI*	163	3	2	AAFI01000022	EAL71051	0185027	[37]
CBP10	*JC2V2_0_03060*	107	0	2	AAFI01000044	EAL68782	0169106	
CBP11	*BC4V2_0_02040*	192	1	4	AAFI01000145	EAL63932	0187233	No EST
CBP12	*BC4V2_0_00355*	171	3	4	AAFI01000093	EAL65589	0185595	
CBP13	*BC4V2_0_00374*	102	1	4	AAFI01000093	EAL65599	0185615	pseudogene?
CBP14	*BC4V2_0_01173*	139	1	4	AAFI01000121	EAL64796	0186418	
Frequenin1	*cbpJ*	194	1	1	AAFI01000011	EAL72455	0231009	[65]
Frequenin2	*cbpK*	192	1	2	AAFI01000027 AY655132	EAL70072 AAT72748	0167922	[65]
Frequenin3	*cbpL*	191	2	5	AAFI01000187	EAL63014	0231012	[65]
Frequenin4	*cbpM*	183	2	4	AAFI01000135	EAL64210	0231014	[65]
Frequenin5	*ncsA*	186	2	2	AAFI01000030	EAL69716	0231007	[65]
Frequenin6	*JC2V2_0_01524*	185	1	2	AAFI01000027	EAL70071	0167921	cluster with *cbpK*
Frequenin7	*JC2V2_0_01529*	225	3	2	AAFI01000027	EAL70283	0217470	
Calfumirin	*cafA*	169	3	3	AAFI01000052	EAL68086	0214954	[66]
CentrinA	*cenA*	151	3	5	AAFI01000171	EAL63239	0219932	
CentrinB	*cenB*	150	1	5	AAFI01000056	EAL67875	0220501	
