# Supplementary figures and images for: Thirteen is enough: the myosins of Dictyostelium discoideum and their light chains
Source: BMC Genomics. 2006 Jul 20;7:183. doi: 10.1186/1471-2164-7-183 (PMC1634994; doi:10.1186/1471-2164-7-183)

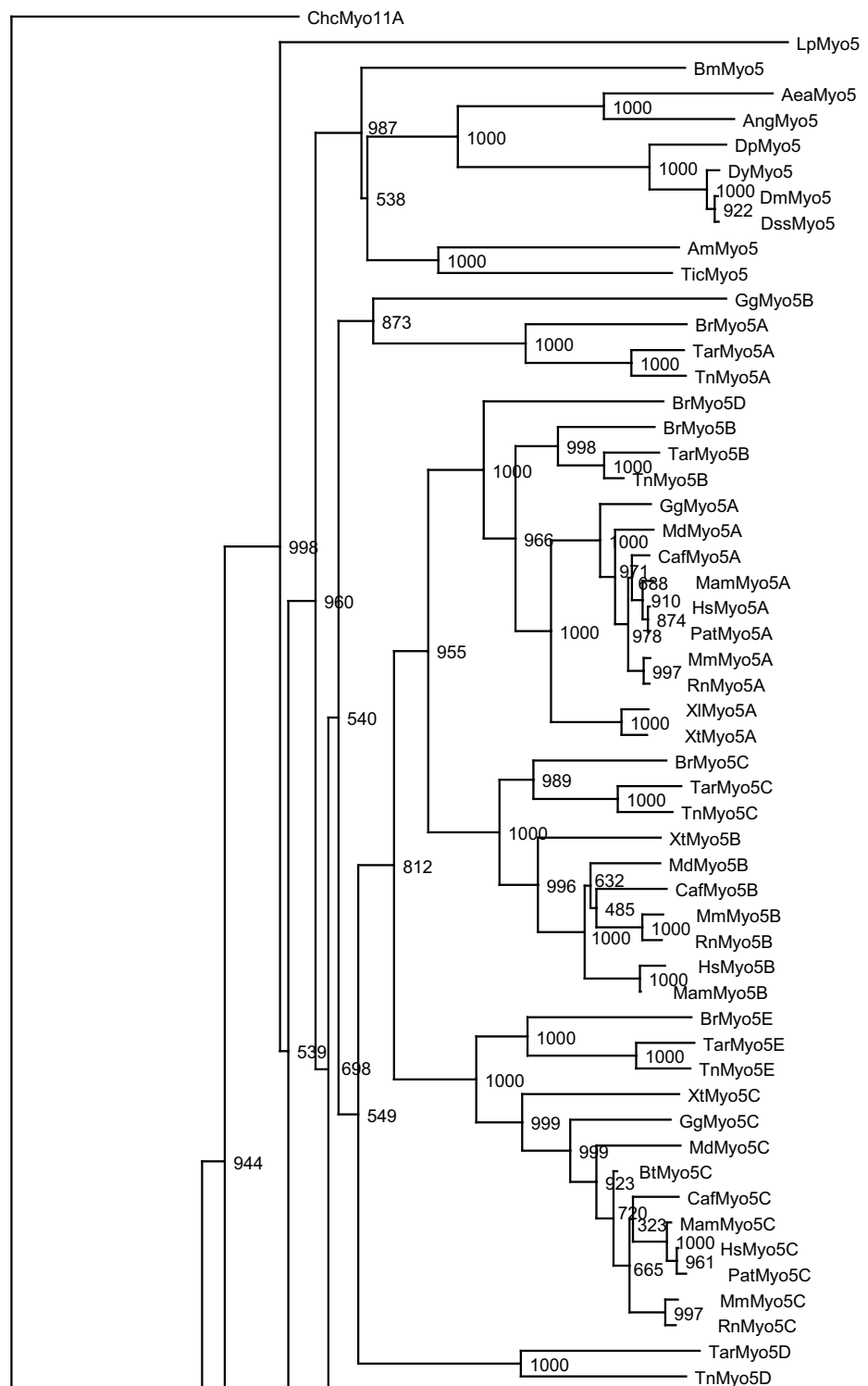

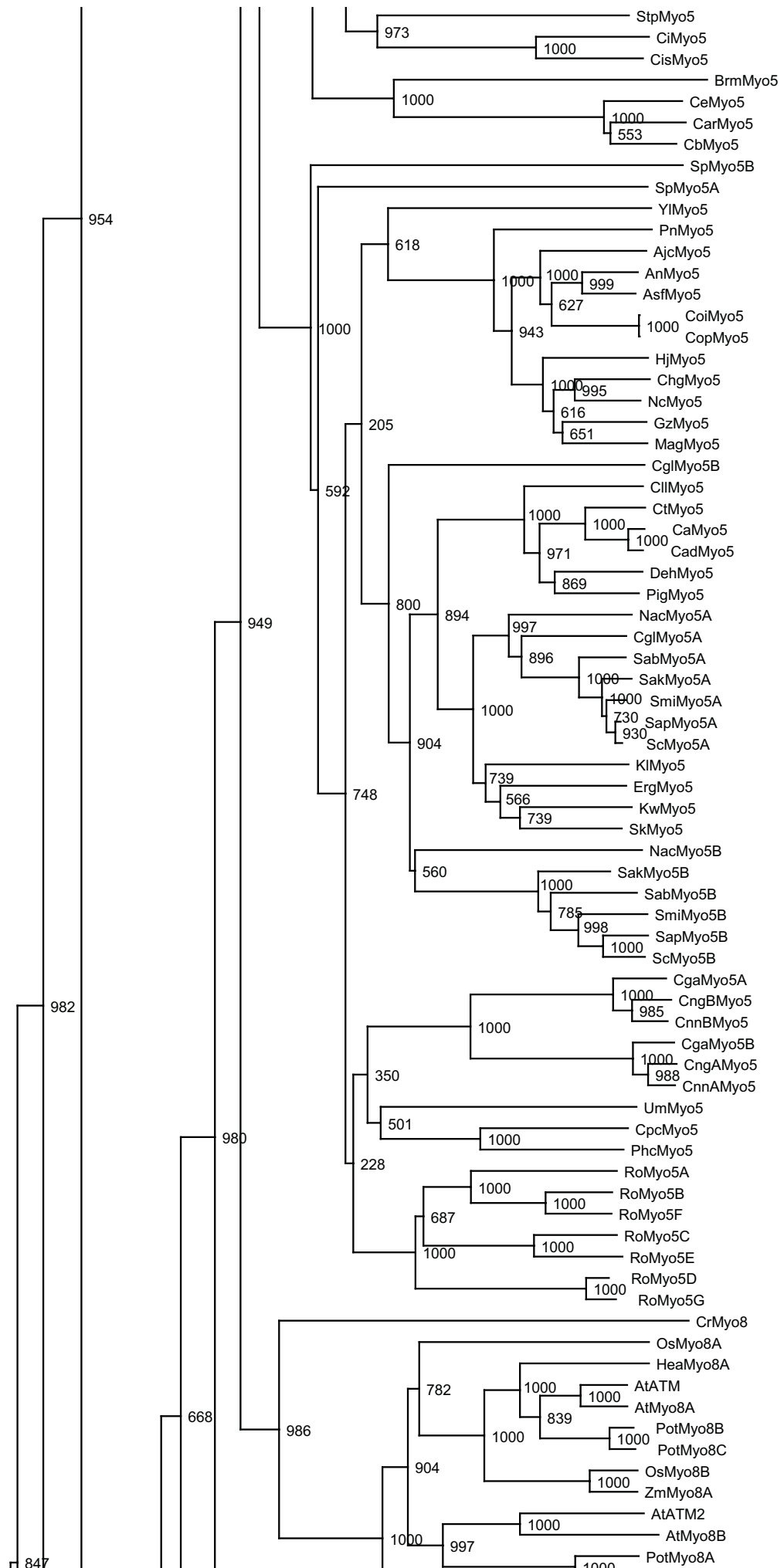

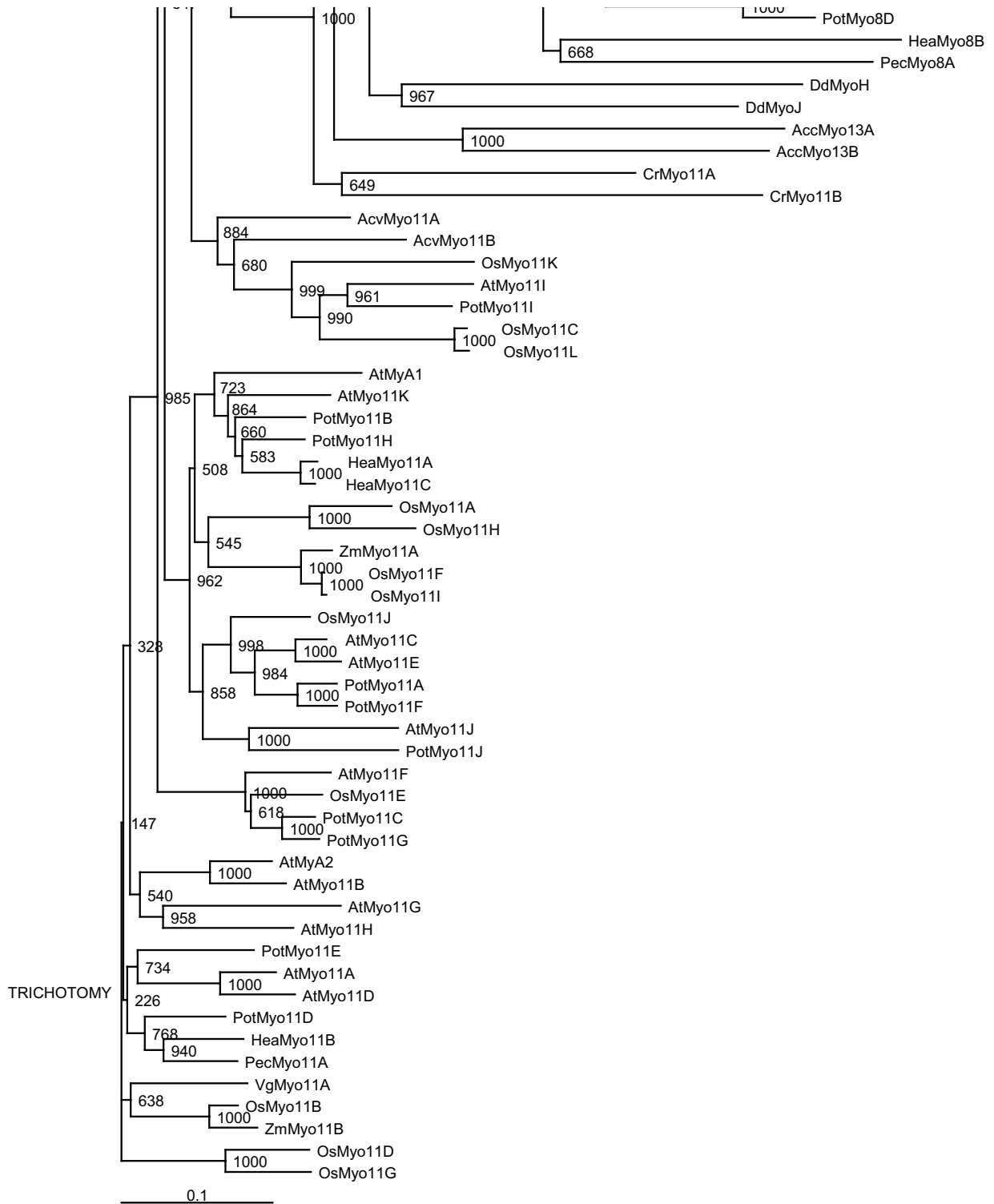

Supplement: Additional File 1 — Complete phylogenetic tree of the class-V, -VIII, and -XI myosins. [file 1471-2164-7-183-S1.pdf]

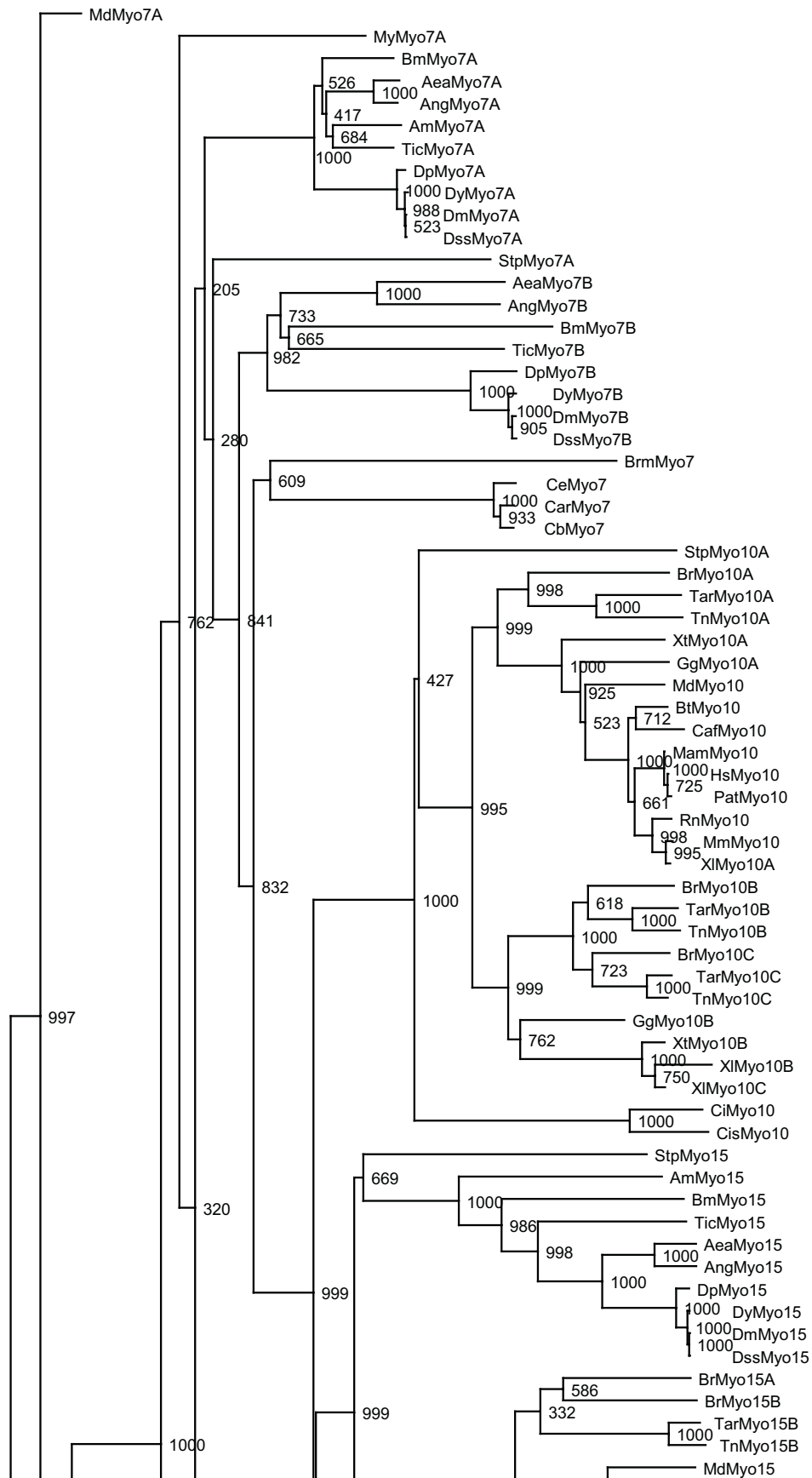

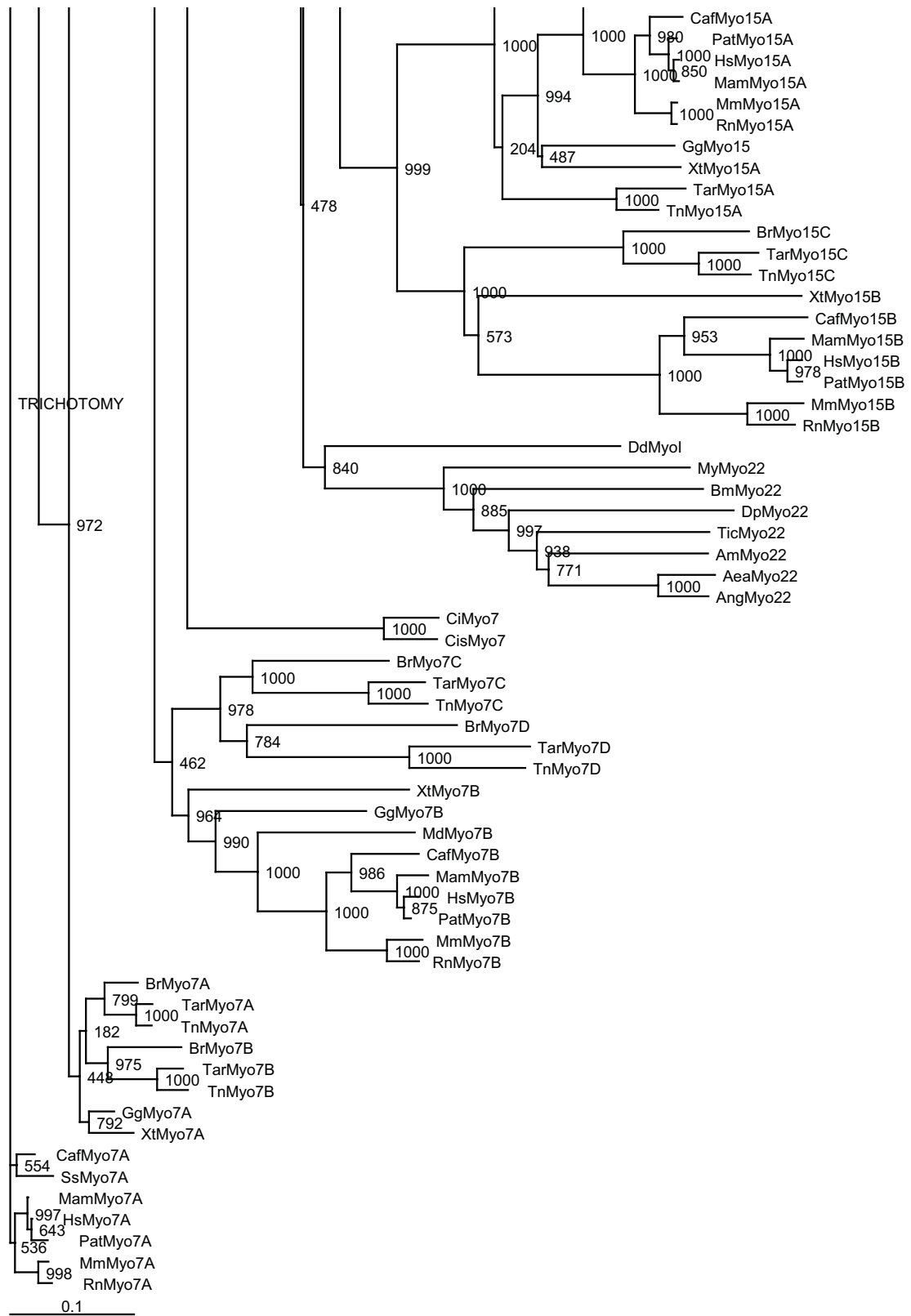

Supplement: Additional File 2 — Complete phylogenetic tree of the class-VII, -X, -XV and -XXII myosins. [file 1471-2164-7-183-S2.pdf]

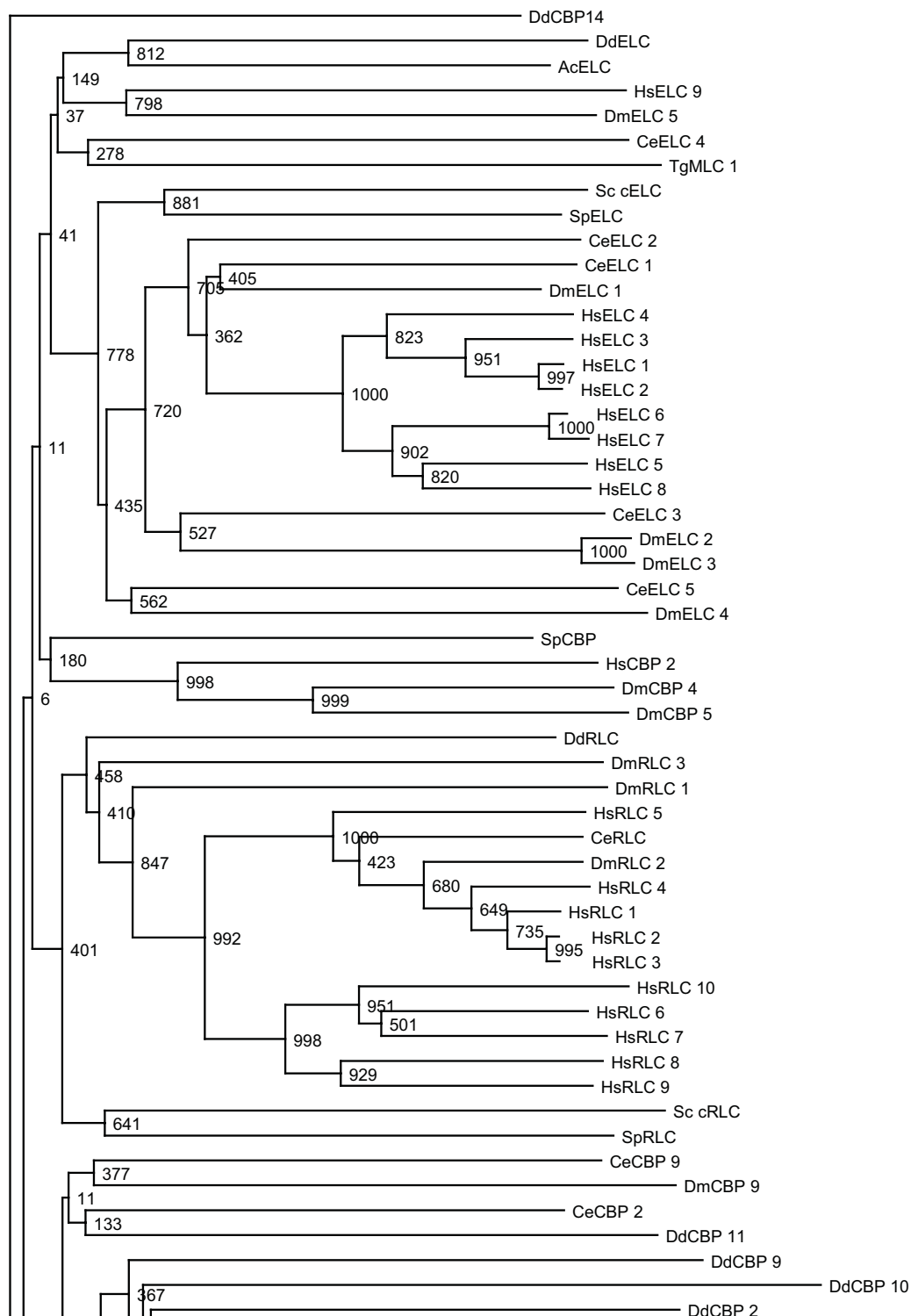

TRICHOTOMY

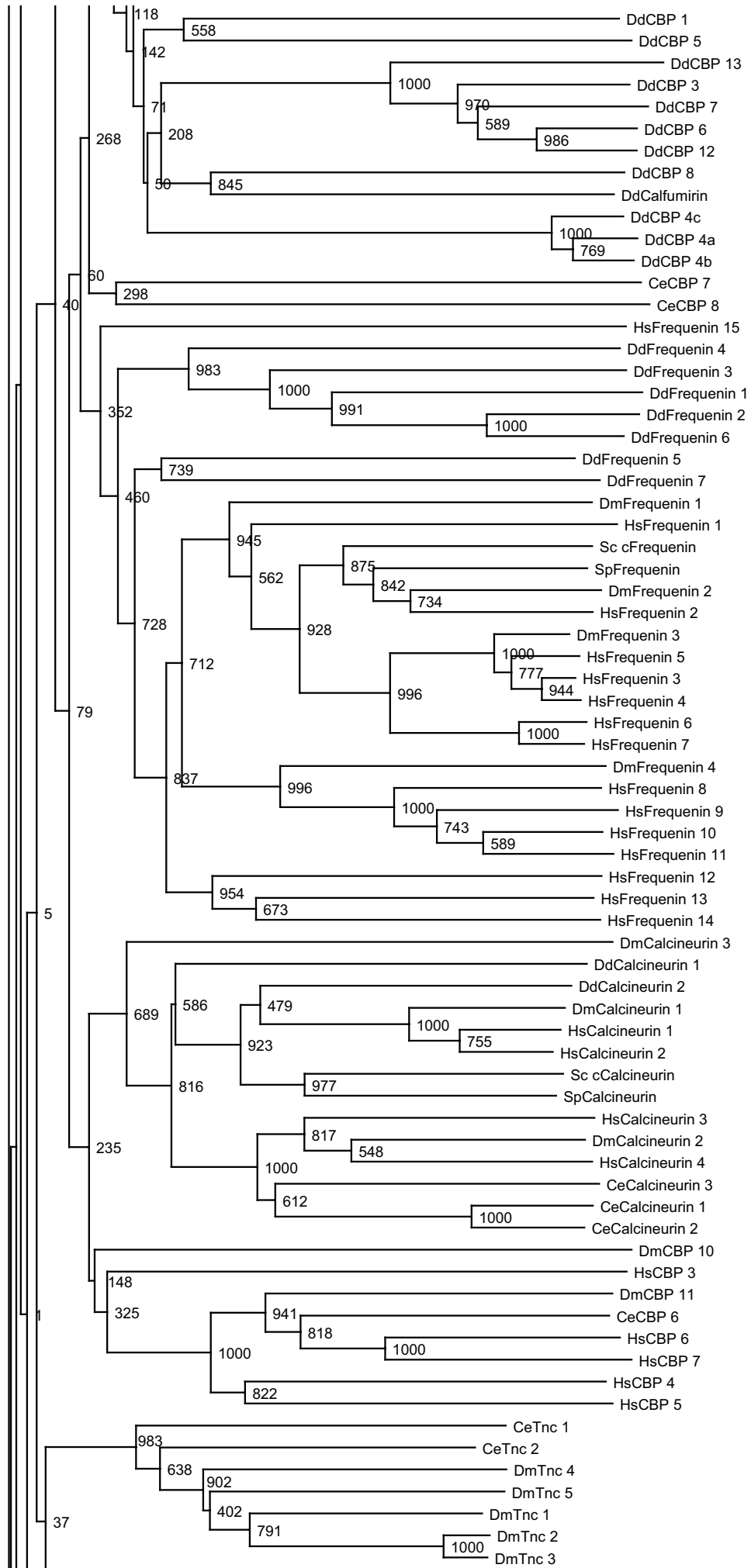

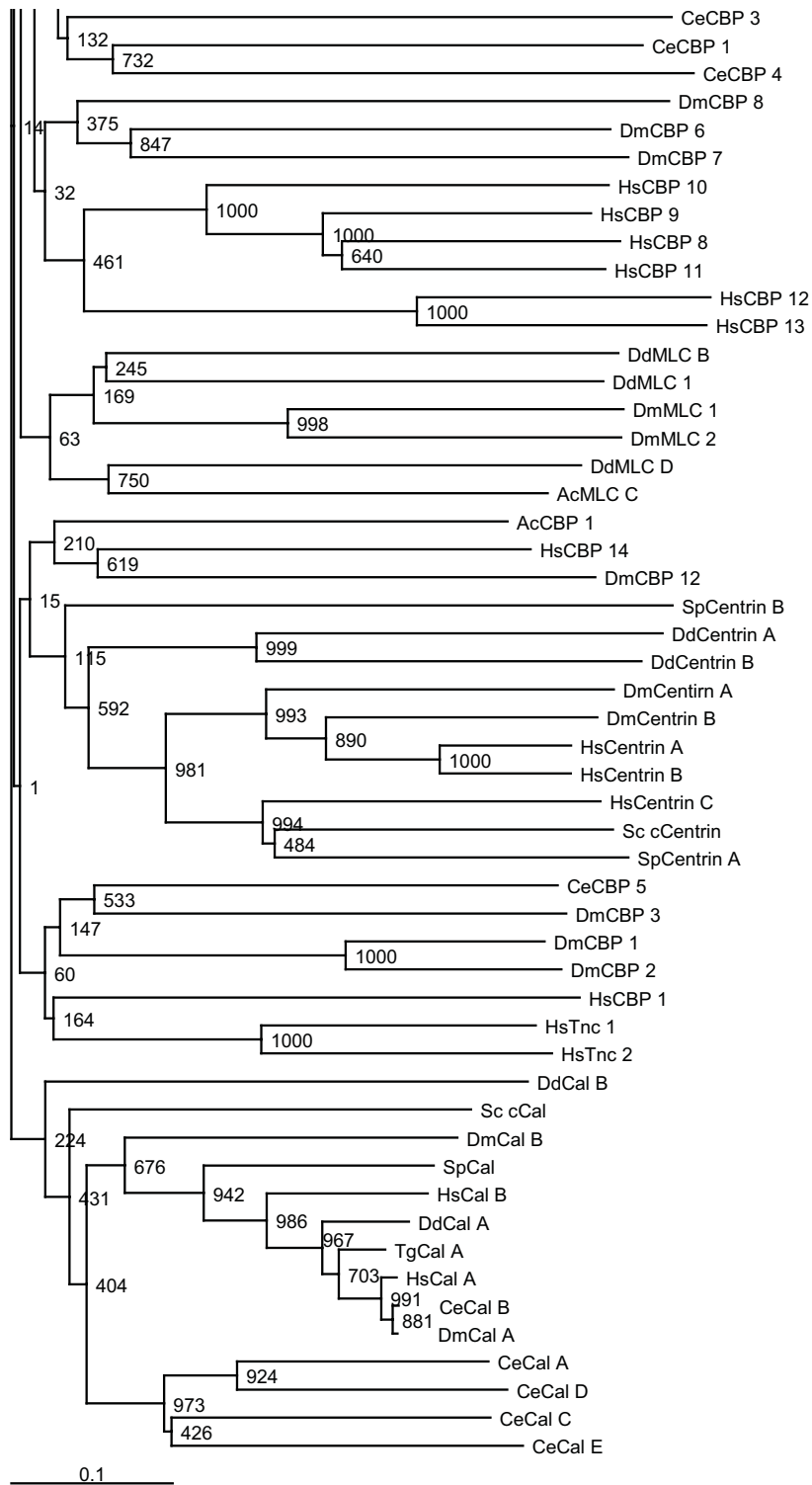

Supplement: Additional File 3 — Complete phylogenetic tree of the calmodulin related proteins. [file 1471-2164-7-183-S3.pdf]
